# Paradox of low field enhancement factor for field emission nanodiodes in relation to quantum screening effects

**DOI:** 10.1186/1556-276X-7-125

**Published:** 2012-02-14

**Authors:** Tsung-Chieh Cheng, Pai-Yen Chen, Shen-Yao Wu

**Affiliations:** 1 Department of Mechanical Engineering, National Kaohsiung University of Applied Science, 415 Chien Kung Road, Sanmin District, Kaohsiung, 80778, Taiwan; 2 Department of Electrical and Computer Engineering, University of Texas at Austin, 2501 Speedway, Austin, TX, 78712, USA

**Keywords:** quantum screening effects, field emission, vacuum electronics, Fowler-Nordheim tunneling, silicon nanostructures

## Abstract

We put forward the quantum screening effect in field emission [FE] nanodiodes, explaining relatively low field enhancement factors due to the increased potential barrier that impedes the electron Fowler-Nordheim tunneling, which is usually observed in nanoscale FE experiments. We illustratively show this effect from the energy band diagram and experimentally verify it by performing the nanomanipulation FE measurement for a single P-silicon nanotip emitter (*Φ *= 4.94eV), with a scanning tungsten-probe anode (work function, *Φ *= 4.5eV) that constitutes a 75-nm vacuum nanogap. A macroscopic FE measurement for the arrays of emitters with a 17-μm vacuum microgap was also performed for a fair comparison.

## Introduction

Recently, micro-/nano-fabricated field emission arrays [FEAs] have attracted a great deal of attention since they have been seen as outstanding electron sources operating with high efficiency, high currents, and fast turn-on times [1-4]. Much effort has been directed toward FEAs' commercial applications in vacuum electronic devices and components, including vacuum lamps and lighting [5], high-power microwave amplifiers [6], thermoelectric cooler [7], microscopes and visualization equipments, parallel e-beam lithography [8] and, of particular interest, the next-generation flat panel displays [9]. With the rapid advent of nanotechnology, various low-dimensional nanomaterials with extreme aspect ratio and high density, like carbon nanotubes [CNTs] [10], zinc oxide nanowires [11], and silicon carbide nanowires [12], have been successfully fabricated with different synthesis methods, and their excellent field emission properties have been widely reported in the literature. It has been known that field emission [FE] properties are highly sensitive to characteristic material properties, like morphology, emitter density, aspect ratio, and electron work function.

Among versatile nanomaterials, silicon nanomaterials are of particular interest due to their excellent compatibility to very-large-scale integration [VLSI] integrated-circuit processes. However, the emission currents from conventional FE cathodes with a large vacuum gap, usually larger than hundred micrometers, are insufficient for practical and realistic electronic applications, and the large operating voltages are far too high for being integrated into the standard CMOS electronic devices. However, following Moore's law for microelectronic devices, within the ever-improving VLSI/USLI technology, nanodiodes with a nanogap may be envisioned in the very near future, and they may bring advantages of lower power consumption and high-current output for the next-generation vacuum nanoelectronics. When aggressively squeezing the vacuum electronic devices to the nanoscale level, many challenging and anomalous physical effects will emerge, such as the space charge effect [13], energy accumulation that increases the burnout resistance, and vacuum sealing; these are, however, ignorable in conventional FE microdevices. The most striking effect, which is, however, largely ignored in previous literature, is the relatively low field enhancement factor obtained from the experimental Fowler-Nordheim [FN] plot. The field enhancement factor may describe the ability of specific emitters to amplify the macroscopic field, which in turn determines the total emission current. In many experiments of nanoscale FE characterizations (usually apply a tungsten [W]-probe anode with its work function, *Φ *= 4.4eV [14]) for a single carbon nanotube [15], nanofiber, or nanoflakes [16] with a work function of *Φ *= 5.1 ∓ 0.1eV, the reported field enhancement factors for nanogaps of *d *= 60 to approximately 380 nm are in the range of 90 to approximately 380 nm [17]. These values are, however, much lower than those obtained from FE microdiodes with a vacuum gap larger than a few hundred micrometers, of which the field enhancement factor is usually in the order of 10^3 ^to 10^4 ^[18]. These values are already conservative, and if we consider a more realistic scenario, the electrostatic screening effect [10] implies that the field enhancement factor for the arrays of nanoemitters should be strongly dependent on the density and arrangement of nanoemitters. It is therefore relevant to explain the anomalously low field enhancement factor in vacuum nanodiodes. For such small vacuum nanogap, the simple electrostatic explanation may not be sufficient, and a careful look into a quantum level is necessary by considering all energy levels of nanodiode structures, including the effect of anode. It is rather illustrative to use the energy band diagram to study how the field enhancement factor may be affected by a quantum effect due to the work function difference between the emitters and anode, especially when the work function of the field emitter (i.e., CNT emitters) is higher than that of the anode (i.e., W-probe) [19].

## Experimental details

**Figure 1:**
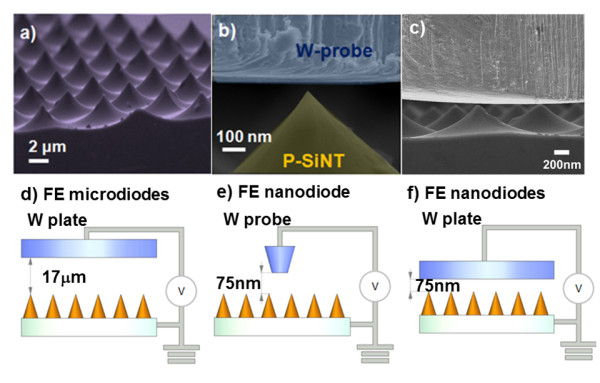
**SEM images and schematic diagram**. SEM images for (**a**) P-silicon nanotip [P-SiNT] arrays, (**b**) tungsten [W]-probe anode and a single P-SiNT emitter, and (**c**) W-plate anode and P-SiNT emitters' array, forming a field emission [FE] nanodiode. Schematic diagram for (**d**) FE microdiodes, (**e**) FE nanodiode with W-probe anode, and (**f**) FE nanodiodes with W-plate anode.

Attempting to validate the quantum effect due to the anode-to-cathode separation at the nanoscale vacuum gap (the so-called quantum screening effect in this paper), we further implement FE experiments on nanodiodes and microdiodes. Here, we specifically equipped the HRSEM system (JEOL JSM-6500F, JEOL Ltd., Akishima, Tokyo, Japan) with a *in situ *nanomanipulation of FE measurement apparatus [20], where the laboratory-prototype vacuum nanodiode is formed by a nanomotor-manipulated W-probe/-plate anode and P-silicon nanotips [P-SiNTs] emitter (see the scanning electron microscopy [SEM] image in Figure 1). The movement of W-probe or -plate attached on the nanomotor is controlled independently from the SEM stage by the three-axes piezo-driven mechanical displacement system, with a step resolution up to ± 0.5 nm in all three axes. Therefore, the height of vacuum gap can be accurately controlled by the nanomotor [21] and, moreover, the direct SEM observation is readily available. In our experiment, the FE was measured at a vacuum level up to 9.6 × 10^-7 ^Torr. A Keithley-237 high-voltage analyzer (Keithley Instruments, Inc., Cleveland, OH, USA) was used as the voltage source, ranging from 0 to 300 V, and then was used to measure the emission current. The large-area, sharpen-end, uniform, and well-defined P-SiNTs were fabricated by a simple three-step process. First, photoresist mask was patterned by anisotropic inductively coupled plasma etching to make high aspect ratio circular rods. Then, isotropic etching was used to produce sharp emitters by an undercutting effect under the mask, with a control proper of plasma conditions. Finally, the silicon nanotips were oxidized in the furnace, and the surface silicon oxide was then removed by wet etching using a buffered oxide etching [BOE] solution to form the P-SiNT array in Figure 1. Figure 1a shows the SEM image of the arrays of P-SiNT emitters. Figure 1b shows a prototyping vacuum nanodiode formed by the nanomotor-controlled W-probe and a single P-SiNT emitter (schematic diagram as shown in Figure 1e), and Figure 1c, W-plate and the P-SiNT emitters' array (schematic diagram as shown in Figure 1f). The W-probe with a flat end and a diameter of 8 μm was fabricated by electrolysis with a KOH solution and flattened by the chemical mechanical polishing [21]. Figure 1d illustrates the macroscopic FE measurement for a FE microdiode, with a 17-μm vacuum gap between the SiNT FEA and the W-plate anode (with an effective area of 50 mm^2^), in some sense similar to the parallel-plate capacitor geometry. Before the FE measurement, the primitive P-SiNTs were treated with the BOE wet etching for 30 s to remove the native oxide and surface contamination that may affect electron emission at the emitter surface.

In order to obtain the exact work function (*Φ*) of SiNTs, which could be very different from the bulk silicon, the scanning Kelvin probe microscopy (NT-MDT Solver P47, NT MDT Co., Zelenograd, Moscow, Russia) [22] was applied to measure the work function (*Φ*) of SiNTs. Here, an AC voltage (75.2 kHz) was first applied to the SiNTs sample, inducing an oscillating electrostatic force between the conductive atomic force microscope tip and the sample. Then, the compensation of electrostatic forces at this frequency was achieved by adjusting a DC bias to match exactly the contact potential difference [CPD] between the tip and the sample. Since the work function of the cantilever is known, the value of CPD can be determined by *Φ*_sample _= *Φ*_cantilever _+ CPD. Here, the measured work function of SiNW emitters is 4.94 eV.

## Results and discussion

**Figure 2:**
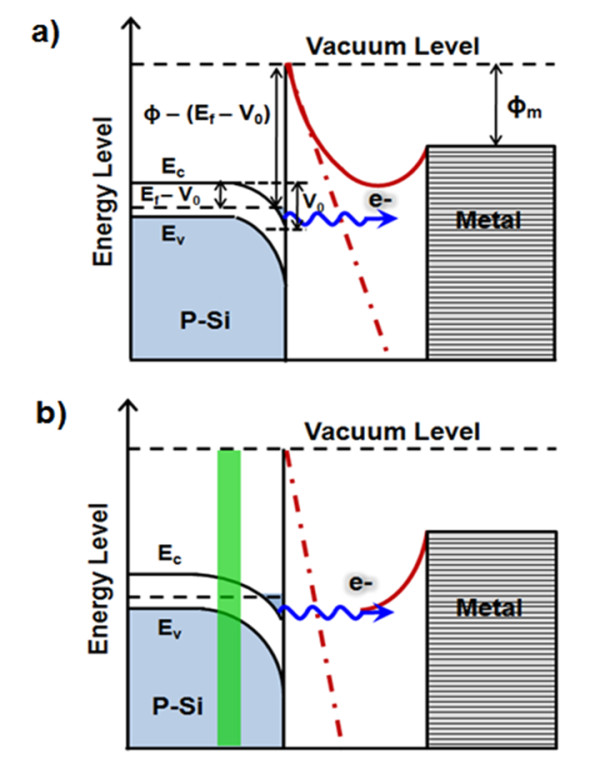
**Energy band diagram of the electron field emission**. (**a**) Normal and (**b**) strong applied electrostatic bias fields in a FE nanodiode. The dashed line shows the potential distribution for a FE microdiode, with a distant anode (not shown here) and a very large anode-to-cathode distance.

**Figure 3:**
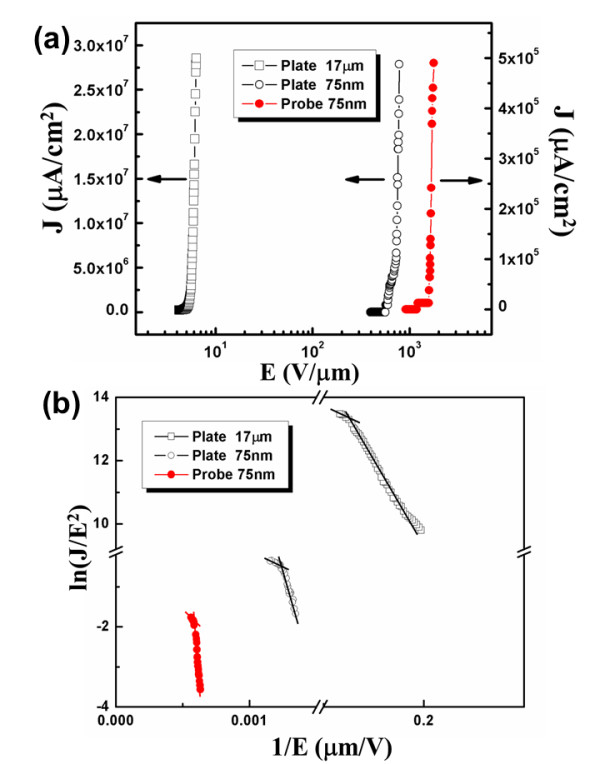
**Field emission properties and FN plot**. Properties of (**a**) the current density versus applied field and (**b**) the corresponding FN plot for a single P-SiNT nanodiode (red circle), P-SiNT array nanodiode (hollow circle), and P-SiNT array microdiode (hollow square).

The field emission from emitters can be described by the well-known FN tunneling, where the emission current (*I*), as a function of the local field (*F*) at the tip surface of emitters, is given by *I*= *C*(*F*^2^/*Φ*)exp(-*BΦ*^3/2^/*F*), where *C *and *B *are constants (*B *= 6.83 × 10^9^VeV^-3/2^m^-1^, obtained from quantum mechanics derivations), and *Φ *is the work function of emitter in eV. The work function (*Φ*) is defined as the lowest energy required for extracting an electron from the surface of a conducting material, such as CNTs or graphene flakes, to a point just beyond the metal surface with zero kinetic energy. In general, the local field (*F*) is related to the applied anode voltage by *F *= *γE*_0 _= *γV*/*D*, where *γ *is the field enhancement factor and *E*_0 _= *V*/*D *is the macroscopic applied electric field (*D *is the distance of W anode to the bottom of emitters as shown in Figure 2). In the scenario of electron field emissions, the FN plot of In (*I*/*V*^2^) versus *V*^-1 ^should fit a straight line with a slope of -6.44 × 10^9^*Φ*^-3/2^*d*/*γ*. For semiconductor field emitters, field penetration into the semiconductor may lead to a change of the carrier concentration in the near-surface region and bending of energy band at the emitter surface, as shown in Figure 2a. In this scenario (Figure 2a), the effective work function (*Φ*_eff_) for electron emission into the vacuum results from the sum of several potential barriers at the surface: *Φ*_eff _= *ϕ*-(*E_f _*- *V*_0_), where *ϕ *is the ionization energy, *E_f _*is the Fermi level, and *V*_0 _is the lowering of the conduction band at the surface due to the field penetration [23]. Figure 3a reports the emission current density (J) (in A/cm^2^) versus the applied field strength (*E*_0_) (in V/μm) for a single P-SiNT that constitutes the FE nanodiode with a nanogap of 75 nm (solid circles), and a FE nano/microdiode consisting of the P-SiNT FEA and W-plate anode with a nanogap of 75 nm (hollow circles) and microgap of 17 μm (hollow squares). We notice that the emission current from each SiNTs in the FEA is statistically uniform, with a standard variation of 13.6% obtained by randomly measuring ten emitters using the nanomanipulation technique. Figure 3b reports the corresponding FN plot; the linearity of FN plots evidently implies the FN quantum tunneling mechanism. Besides, Table 1 summarizes the field emission properties of P-SiNT emitters with W-probe or -plate anode with different separation. The results indicated that the field enhancement and turn-on field of P-SiNTs FEA with nanogap separation is larger than with microgap separation.

**Table 1:** Field emission properties of P-SiNT emitter with W-probe or -plate anode at different separations

	Anode-to-cathode distance	Enhancement factor	Turn-on field (V/μm, at *J *= 10 mA/cm^2^)
W-probe anode	75 nm	3.17	1,286
W-plate anode	75 nm	436	603
W-plate anode	17 μm	616.2	4.617

In order to explain our experimental results, a quantum screening effect model is proposed here. A schematic diagram about energy band distribution in the vacuum gap is shown in Figure 2. In the vacuum region in Figure 2b, the dashed lines represent the potential contours for a FE *microdiode *with the anode located at a long distance (usually larger than several micrometers and not shown in this figure) such that its influence on electron emissions in the near-field region of emitter surface may be neglected. The solid line in Figure 2a represents the one for a very short anode-to-cathode distance, where the potential barrier seen by an electron tunneling through a vacuum gap is dramatically increased; thus, an electron may need more energy or a higher applied field for tunneling through the potential barrier compared with electron emission at a large anode-to-cathode distance. From Figure 3a, it is quite evident that the microdiode requires much lower turn-on field due to the absence of the quantum screening effect. As a result, except for the electrostatic screening effect depending on the emitter's density and sharpness, the vacuum gap height is also important when considering the effect of potential barrier on the transport of electrons.

It is interesting to note that for high applied fields, the flattening of FN plot (also known as the saturation effect) is observed. Figure 3b is similar to Figure 3a, but for a higher electrostatic bias field, showing that the conduction band of p-type field emitter will be degenerated at the surface, and the green shaded region in Figure 2b indicates a depletion region between the *p-type interior *and the n-type surface, of which the Fermi level lies in the middle of the energy gap. This leads to a minimum concentration of electrons and holes in such region, in some sense similar to the reverse-biased condition in a p-n junction [24]. We also notice that due to the space charge in the vacuum region [13], even the applied field is very strong, potential barrier at the surface remains much higher than that of microdiodes, and therefore, the quantum screening effect is hardly eased. In the linear region (before the saturation occurs) of FN plot, the field enhancement factors calculated from the slope of FN plot are respectively 436 and 616.2 for the nanodiode and the microdiode with W-plate from our experimental results, as have been observed in many nanoscale FE measurements. As described in previous papers [3,5,19], the field enhancement factor is usually defined as the local field (*F*) over the applied field (*E*_0_), where the applied field is usually taken as the applied voltage over anode to the bottom of cathode separation *D *(as shown in Figure 2), or *γ = F/E*_0_, where *E*_0 _*= V/D*. According to our quantum screening effect model, as anode to the top of cathode separation *d *(like the top of our P-SiNT) approaches to nano-distance, the local field will decrease because of the quantum screening effect, but the applied field did not decrease because the separation of anode to the bottom of cathode is still large. It is quite evident that the quantum screening effect may decrease the field enhancement factor. Besides, comparing with the field emission properties of SiNTs with W-probe and with W-plate, the results showed that the enhancement factor of SiNTs with W-plate is larger than with W-probe, and the turn-on field of SiNTs with W-plate is less than with W-probe. In our experiment, the SiNTs array is not too dense so that the SiNT emitters' array with W-plate anode geometry can be served as a parallel circuit without considering the space charge effect so that it can decrease the turn-on field. Besides, according to Wang's model [25], more electron emission emitters with certain density will increase the field enhancement factor, so the SiNT emitters' array with W-plate have larger field enhancement factor than the SiNT emitters' array with W-probe in this paper.

## Conclusion

In summary, we have experimentally demonstrated that in FE nanodiodes, the quantum screening effect may significantly increase the turn-on field and reduce the field enhancement factor and, therefore, deteriorate the electron emission efficiency. Furthermore, the experimental evidence is supported by a simple band diagram analysis. This quantum screening effect, which describes the relatively low field enhancement factors and higher turn-on field in most nanoscale FE experiments, is particularly crucial for vacuum nanoelectronic devices.

## Competing interests

The authors declare that they have no competing interests.

## Authors' contributions

TC conducted and finished the idea and experiments. SW helped and supervised TC for the experiments. PC helped TC to modify the quantum screening effect concept. TC and PC revised and edited the manuscript. All authors read and approved the final manuscript.
